# L-Tetrahydropalmatine synergizes cytotoxic CD8^+^ T mediated antitumor and ferroptosis in gastric cancer

**DOI:** 10.1038/s41420-025-02825-x

**Published:** 2025-11-24

**Authors:** Le Zhou, Yaqin Wei, Kai Lin, Yu Lu

**Affiliations:** Department of Anesthesiology, Sichuan Provincial People’s Hospital, University of Electronic Science and Technology of China, Chengdu, China

**Keywords:** Immune evasion, Cancer microenvironment

## Abstract

L-Tetrahydropalmatine (L-THP) is an isoquinoline alkaloid derived from the traditional Chinese herb Corydalis (*genus Corydalis*), particularly its analgesic and sedative effects. The in-depth function of L-THP on gastric cancer (GC) immune escape is still unclear. Here, the present research aimed to investigate the function of L-THP on GC antitumor response. Results indicated that L-THP promoted the cytotoxicity and antitumor response of CD8^+^ T cells to GC cells. Moreover, L-THP up-regulated the ferroptosis of GC cells. Mechanistically, L-THP recognized the FSP1 protein and bound to FSP1 with high binding affinity. L-THP reduced the FSP1 stability to trigger the ferroptosis of GC cells. In conclusion, this study shows that L-THP synergizes cytotoxic CD8^+^ T cell-mediated GC antitumor response and ferroptosis through FSP1, providing potent evidence for GC immunoregulation.

## Introduction

Gastric cancer (GC) functions as a major malignancy in human digestive system tumors, ranking as the fourth occurrence cancer type [1, 2]. Every year, more than one million new diagnosed GC cases are reported worldwide, which induces thousands of deaths. Currently, the major management and therapy of GC are composed of surgery, radiotherapy and chemotherapy [3]. The latest researchers focus on immunotherapy and targeted therapy. Nevertheless, despite the great progress has gained for GC treatment, the five-year survival rate of GC patients is still lower than 30% [4]. Thus, it is urgent to discovery the understating for GC development.

Levo-Tetrahydropalmatine (L-THP, also known as Rotundine) is a natural tetrahydro protoberberine isoquinoline alkaloid in traditional Chinese medicine [5, 6]. L-THP is extracted from *corydalis* and *stephania*. L-THP exhibits diverse pharmacological activities, particularly notable for its analgesic and sedative effects [7, 8]. For example, L-THP ameliorates hepatic steatosis in Nonalcoholic fatty liver disease via switching lipid metabolism through the AMPK/SREBP-1c/Sirt1 axis [9]. In hepatocellular carcinoma, the L-THP significantly reduces the mitochondrial membrane potential and proliferation index, as well as the mitochondrial respiration of ATP production, mitochondrial basal respiration, and maximal respiration [10]. However, the in-depth functions of L-THP on GC are mysterious and confusing, which might bring new treatment guidelines.

Herein, our research aimed to determine the function of L-THP in regulating GC immune microenvironment. L-THP blocks the immune evasion of GC cells as an anti-oncogenic factor. This study shows that L-THP synergizes cytotoxic CD8^+^ T cell-mediated GC antitumor response and ferroptosis through FSP1 pathway, providing potent evidence for immunoregulation.

## Results

### L-THP repressed the proliferation of GC cells

**Fig. 1:**
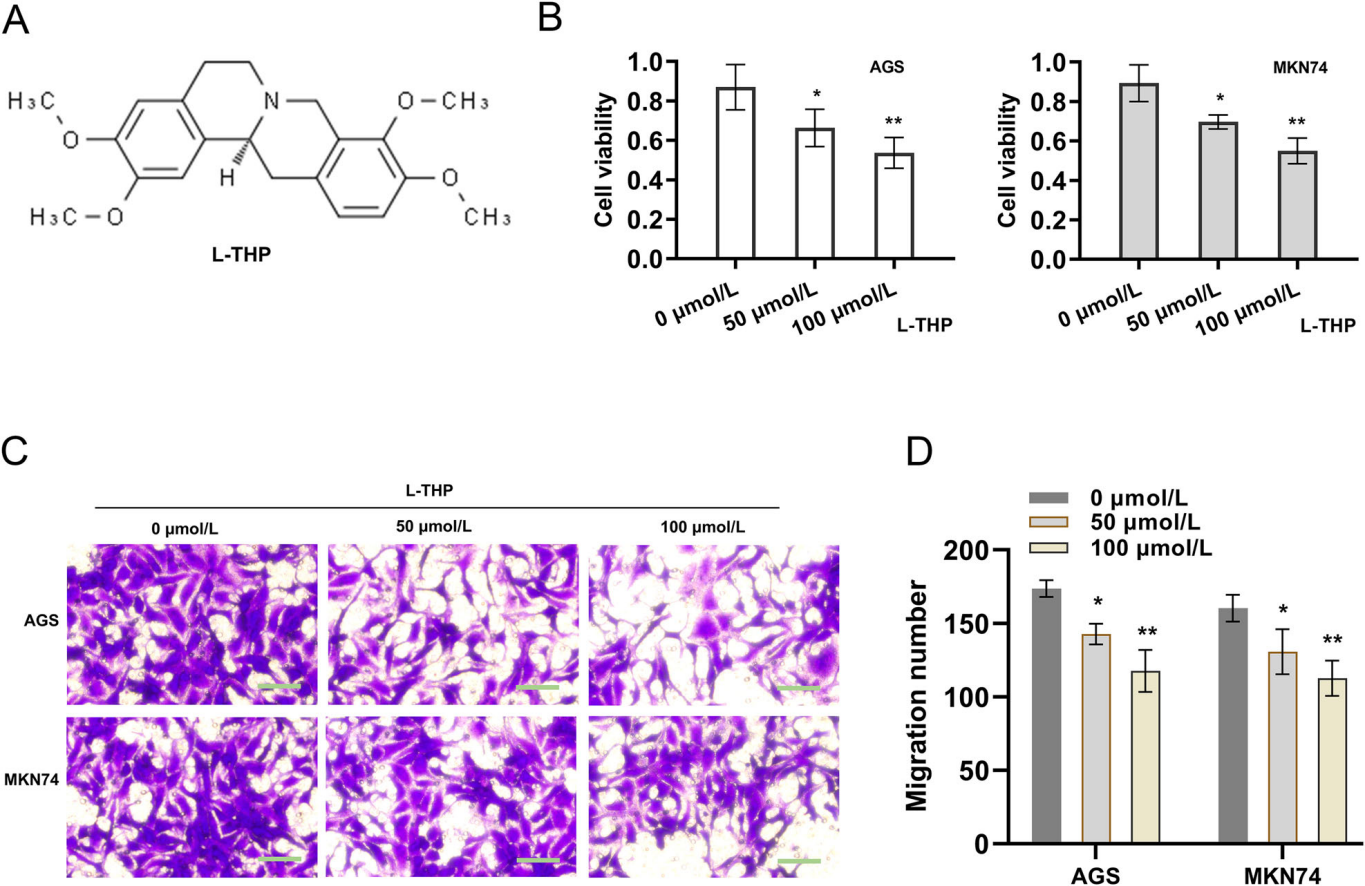
L-THP repressed the proliferation of GC cells. **A** The chemical formula of L-THP (C_21_H_25_NO_4_, molecular weight: 355.44). **B** The proliferative viability of GC cells (AGS, MKN74) was detected by CCK-8 assay. L-THP treatment was performed to GC cells in a concentration-dependent manner (0 μmol/L, 50  μmol/L, 100 μmol/L). **C**, **D** Migration analysis by transwell assay found the migrative viability of GC cells with L-THP concentration-dependent treatment. Bar = 100 μm.*p < 0.05, **p < 0.01.

Previous reports have indicated that L-THP could participate in the oncotherapy through repressing the tumorigenesis. In present research, our assays tested the function of L-THP on GC cells’ viability (Fig. 1A). Result indicated that L-THP could repressed the proliferative viability of GC cells in a concentration-dependent manner (Fig. 1B). Besides, the migration analysis found that L-THP could repressed the migrative viability of GC cells in a concentration-dependent manner (Fig. 1C, D). Therefore, these findings illustrated that L-THP repressed the proliferation of GC cells.

### L-THP accelerated the ferroptosis of GC cells

**Fig. 2:**
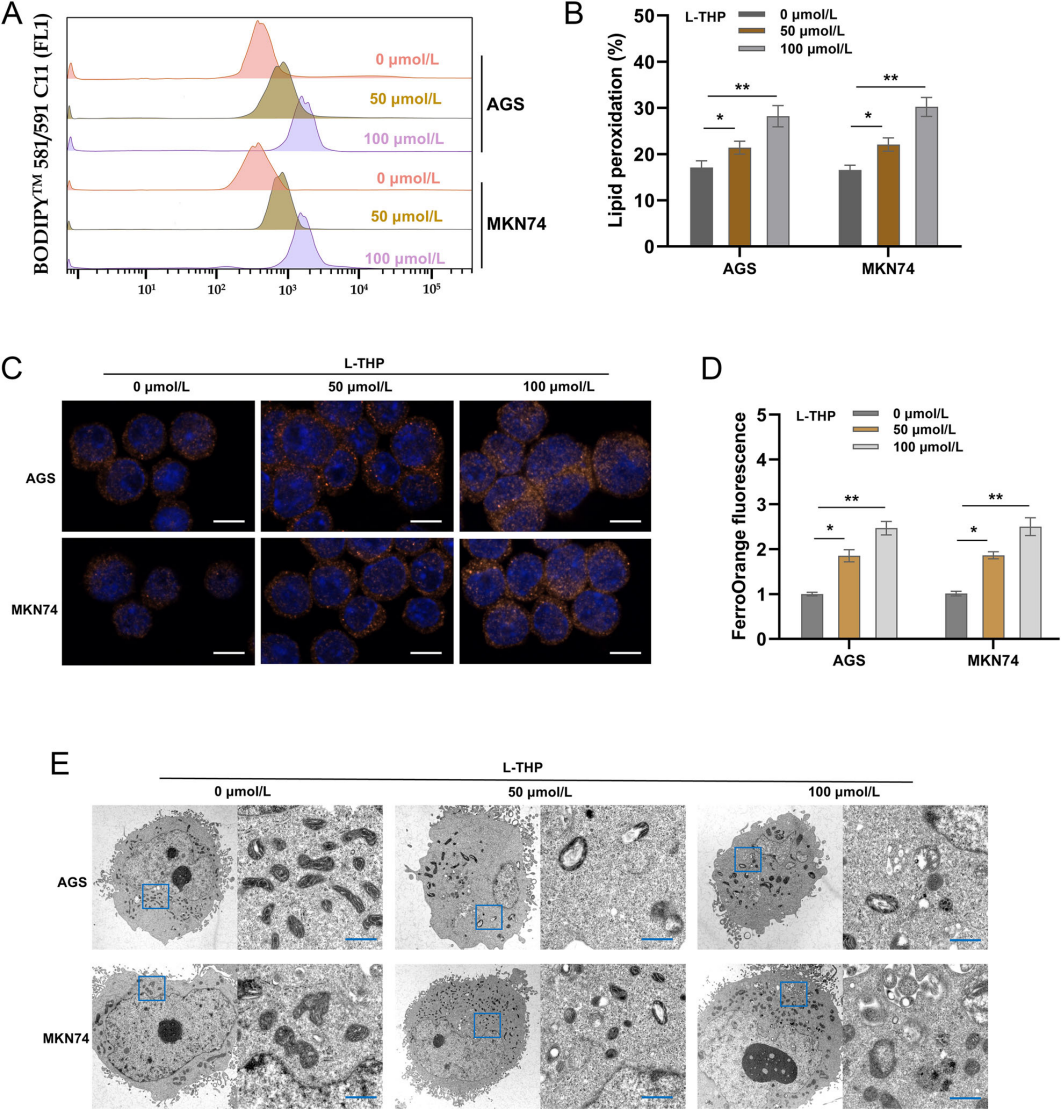
L-THP accelerated the ferroptosis of GC cells. **A**, **B** Lipid peroxidation was detected for quantitative analysis using flow cytometry confocal imaging in GC cells (AGS and MKN74) with L-THP treatment. **C**, **D** FerroOrange fluorescent probe for iron ion concentration (Fe^2+^) was detected in GC cells (AGS and MKN74) with L-THP treatment. **E** TEM analysis revealed the disruption of cellular microstructure in GC cells with L-THP treatment. Bar = 500 nm. *p < 0.05, **p < 0.01.

Our data also found that L-THP could regulate the ferroptosis of GC. Because ferroptosis was driven by the accumulation of lipid peroxidation products in cells, lipid peroxidation was detected for quantitative analysis using flow cytometry confocal imaging. Lipid peroxidation analysis indicated that L-THP treatment increased the lipid peroxidation expression in GC cells (Figs. 2A, B). The intracellular iron ion (Fe^2+^) concentration analysis by FerroOrange fluorescent probe revealed that L-THP treatment up-regulated the Fe^2+^ concentration in GC cells (Figs. 2C, D). Finally, the TEM analysis revealed that L-THP treatment promoted the disruption of cellular microstructure (Fig. 2E). Therefore, these findings illustrated that L-THP accelerated the ferroptosis of GC cells.

### L-THP impaired the GC immune escape through enhancing CD8^+^ T cells’ antitumor response

**Fig. 3:**
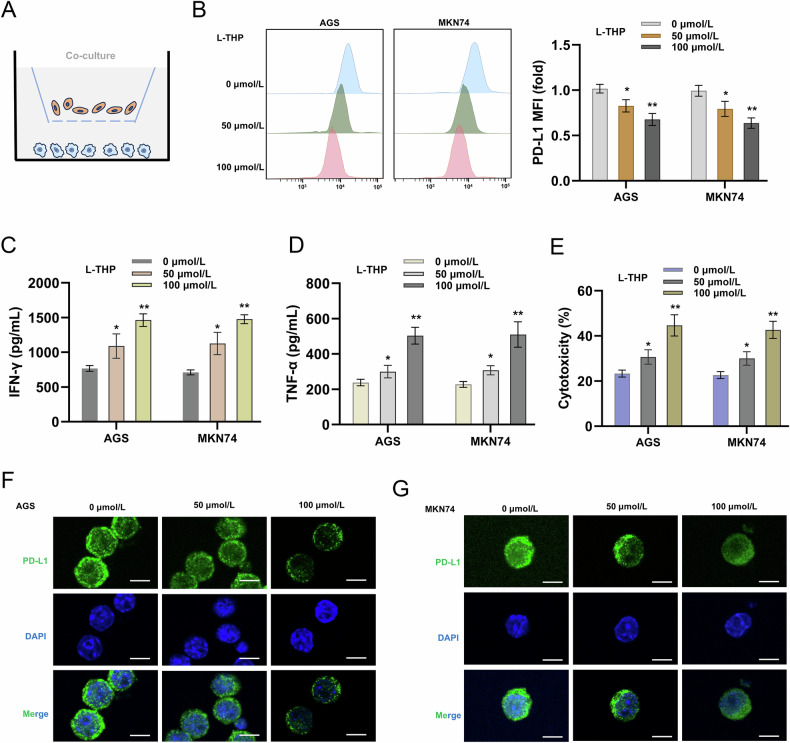
L-THP impaired the GC immune escape through enhancing CD8^+^ T cells’ antitumor response. **A** The in vitro co-culture system of CD8^+^ T cells and GC cells was constructed. Effector CD8^+^ T cells were co-cultured with indicated target GC cells (AGS and MKN74). The L-THP (0 μmol/L, 50 μmol/L, 100 μmol/L) was added to the co-culture of CD8^+^ T cells and GC cells. **B** The cell surface PD-L1 expression in GC cells was detected by flow cytometry. Mean fluorescence intensity (MFI) of GC cell surface PD-L1 in GC cells was calculated in L-THP group versus control group. **C** The secreted level of IFN-γ and **D** TNF-α from co-cultured CD8^+^ T cells were detected by ELISA assay after 48 h co-incubation. **E** The cytotoxicity or killing effect of CD8^+^ T cells towards GC cells was examined using LDH assay. **F**, **G** Immunofluorescence revealed the PD-L1 expression in GC cells. Bar = 10μm. *p < 0.05, **p < 0.01.

In the initial assay, our work detected the potential role of L-THP on GC cells tumorigenesis. Then, to test the role of L-THP on CD8+T cells-mediated GC immune escape, the in vitro co-culture system of CD8+T cells and GC cells was constructed (Fig. 3A). The cell surface PD-L1 expression in GC cells was detected by flow cytometry, and results indicated that L-THP reduced the GC cell-surface PD-L1 expression (Fig. 3B). The levels of secreted IFN-γ and TNF-α from co-cultured CD8^+^ T cells to GC cells were detected, and results indicated that L-THP promoted the secretion of IFN-γ and TNF-α from CD8^+^ T cells, thereby alleviating GC cell-mediated immune suppression (Fig. 3C, D). Cytotoxicity analysis using lactate dehydrogenase (LDH) release quantitative analysis revealed that L-THP up-regulated the LDH release in co-culture supernatants of CD8^+^ T cells and GC cells, indicating the enhancive cytotoxicity of CD8^+^ T cells to GC cells (Fig. 3E). Moreover, the PD-L1 expression in GC cells was detected by immunofluorescence, and results indicated that L-THP reduced the GC cells’ PD-L1 expression (Fig. 3F, G). Therefore, these findings illustrated that L-THP impaired the GC immune escape through enhancing CD8^+^ T cells’ antitumor response.

### FSP1 acted as the target of L-THP

**Fig. 4:**
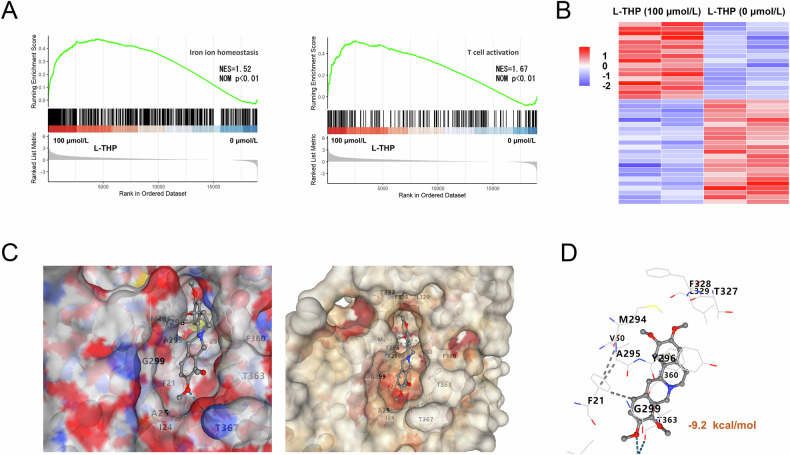
FSP1 acted as the target of L-THP. **A** GSEA of regulated genes in GC cells with L-THP compared with control. **B** RNA-seq analysis reveals the DEGs in GC cells from two groups: control (0 μmol/L), L-THP (100 μmol/). **C**, **D** The L-THP-FSP1 interaction sites in the pocket with a high binding energy (−9.2 kcal/mol). *p < 0.05, **p < 0.01.

Given that our previous finding revealed the negative regulation of L-THP on GC cells’ ferroptosis and antitumor response, our nest work tended to focus on how L-THP mediated the pathophysiology. Firstly, the gene set enrichment analysis (GSEA) confirmed that ferroptosis and T cells activation were both up-regulated by L-THP treatment (Fig. 4A). The genes with differential expression were shown with heatmap of RNA-Seq (Fig. 4B). Molecular docking revealed that L-THP strongly bound to residues FSP1’s G299 and Y296 by hydrogen bonds (Fig. 4C) with a high binding energy (−9.2 kcal/mol) (Fig. 4D). Therefore, these findings illustrated that FSP1 acted as the target of L-THP via molecular binding.

### L-THP impaired FSP1 expression

**Fig. 5:**
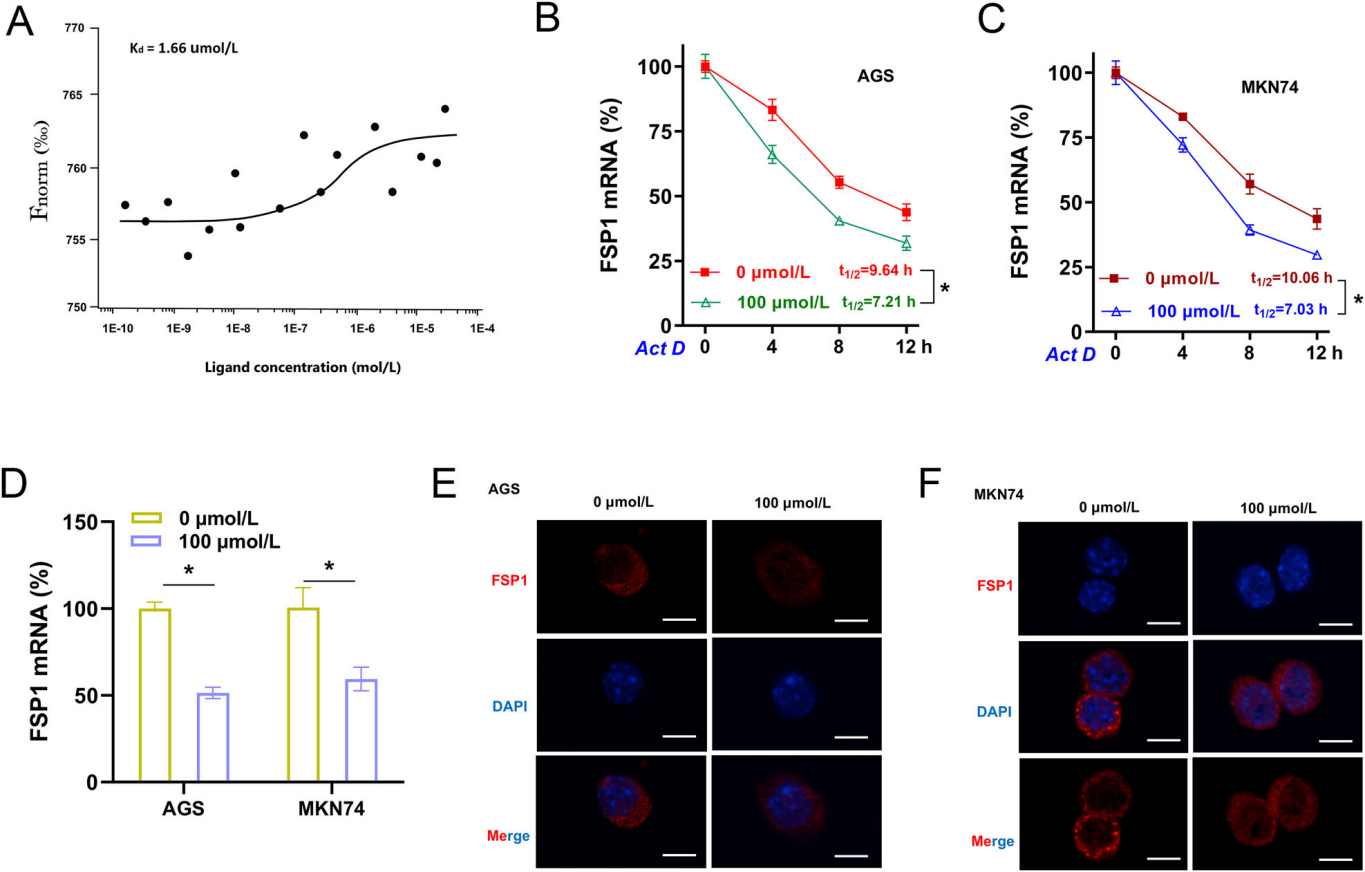
L-THP impaired FSP1 expression. **A** L-THP-FSP1 interaction was confirmed by MST technology. The binding affinity was calculated from the curves indicate. The equilibrium dissociation constant (Kd) was shown in the panel. **B**, **C** RNA decay analysis was performed to test FSP1 mRNA remaining level in GC cells upon Act D preconditioning. GC cells were treated with/without L-THP treatment. **D** RT-PCR indicated the FSP1 mRNA in GC cells with L-THP treatment (0 μmol/L, 100 μmol/). **E**, **F** Immunofluorescence analysis revealed the FSP1 level in GC cells. Bar = 10 μm. *p < 0.05, **p < 0.01.

More evidence is emerging of the roles of ferroptosis and antitumor response on numerous tumorigenesis, and L-THP has shown the tumor suppressor effects. Thus, our work explained the inhibitory effect of L-THP on GC tumor. To further validate the function of L-THP and its depth mechanism on GC tumor immunology, the following assays were performed to confirm this FSP1-dependent manner. The L-THP/FSP1 interaction was further confirmed by a microscale thermophoresis (MST) assay. The equilibrium dissociation constant (Kd) value was 1.66 μmol/L (Fig. 5A). The result indicated a moderately strong binding affinity within L-THP and FSP1. RNA decay analysis revealed that L-THP decreased the FSP1 mRNA remaining level in GC cells upon Act D preconditioning (Fig. 5B, C). Moreover, L-THP also reduced the FSP1 mRNA level in GC cells (Fig. 5D). Immunofluorescence analysis revealed L-THP inhibited the FSP1 level in GC cells (Fig. 5E). Therefore, these findings illustrated that L-THP impaired FSP1 expression in GC cells.

### L-THP triggered GC cells’ ferroptosis and antitumor response through FSP1-dependent manner

**Fig. 6:**
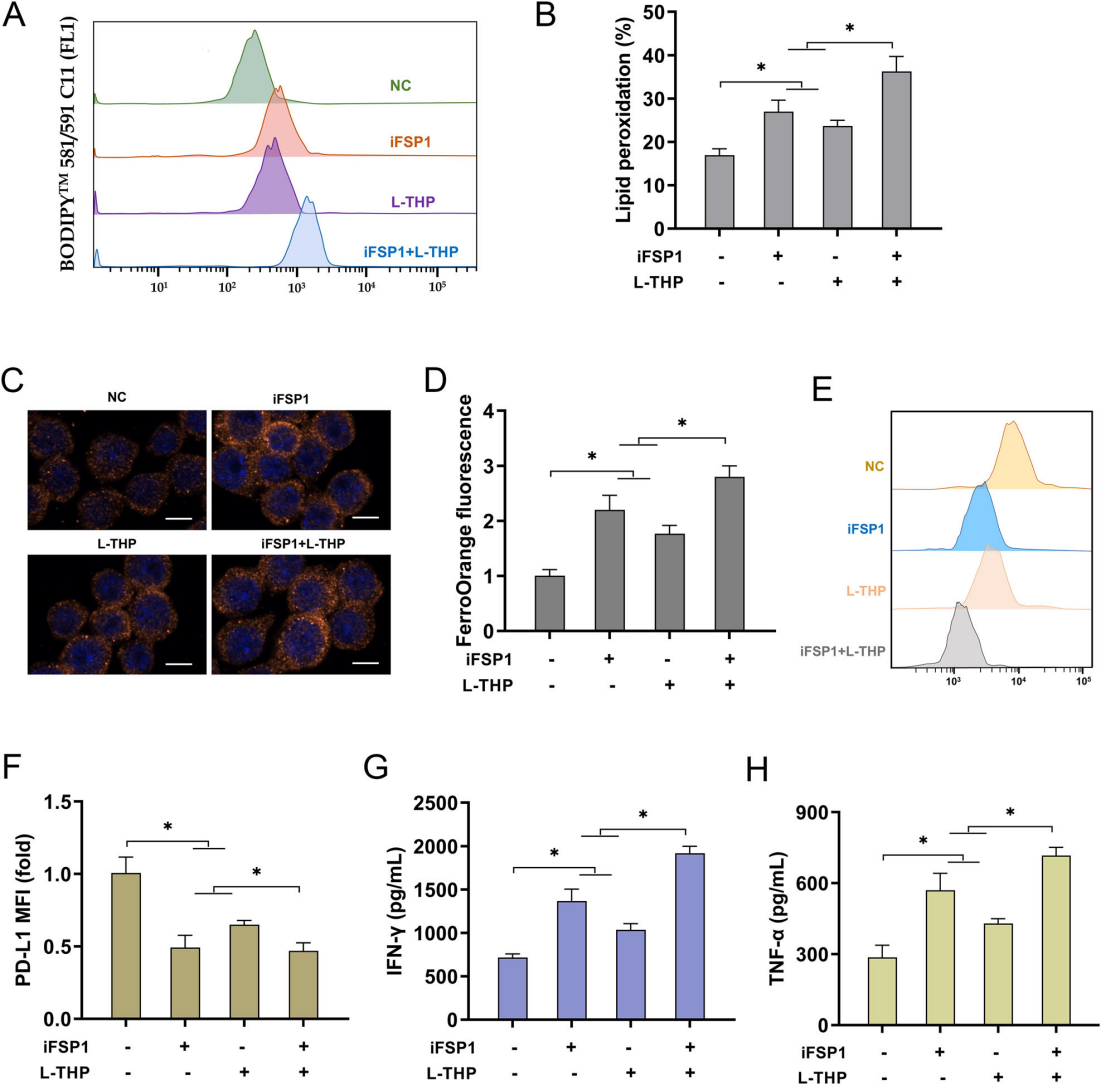
L-THP triggered GC cells’ ferroptosis and antitumor response through FSP1-dependent manner. **A**, **B** The cell surface PD-L1 expression in GC cells was detected by flow cytometry. GC cells (AGS) were treated with iFSP1 (FSP1 selective inhibitor, 5 μM) or with/without L-THP treatment (100 μmol/L). **C**, **D** FerroOrange fluorescent probe for iron ion concentration (Fe^2+^) was detected in GC cells (AGS) with iFSP1 (FSP1 selective inhibitor, 5 μM) or L-THP treatment (100 μmol/L). Bar = 10 μm. **E**, **F** The cell surface PD-L1 expression in GC cells was detected by flow cytometry. Mean fluorescence intensity (MFI) of GC cell surface PD-L1 in GC cells was calculated. **G** The secreted level of IFN-γ and **H** TNF-α from co-cultured CD8^+^ T cells were detected by ELISA assay after 48 h co-incubation. *p < 0.05.

To further validate the function of L-THP and its depth mechanism on GC tumor immunology, the rescue assays were performed to confirm this FSP1-dependent manner. The cell surface PD-L1 expression in GC cells was detected by flow cytometry, and results indicated that iFSP1 (FSP1 selective inhibitor) significantly decreased the surface PD-L1 level in GC cells, and L-THP exerted the same roles (Fig. 6A, B). Besides, their combined efforts enhanced this effect. The intracellular iron ion (Fe^2+^) concentration analysis by FerroOrange fluorescent probe revealed that iFSP1 significantly up-regulated the Fe^2+^ concentration in GC cells, and L-THP exerted the same roles (Fig. 6C, D). The combined efforts of iFSP1 and L-THP enhanced this effect. The cell surface PD-L1 expression in GC cells indicated that iFSP1 significantly down-regulated the GC cell-surface PD-L1 expression, and L-THP exerted the same roles (Fig. 6E, F). And, the synergy efforts of iFSP1 and L-THP enhanced this down-regulation. The levels of secreted IFN-γ and TNF-α from co-cultured CD8^+^ T cells to GC cells were detected, and results indicated that iFSP1 significantly promoted the IFN-γ and TNF-α levels, and L-THP exerted the same roles (Fig. 6G, H). Their synergy enhanced this effect. Therefore, these findings illustrated that L-THP triggered GC cells’ ferroptosis and antitumor response through FSP1-dependent manner.

### L-THP repressed the GC tumor growth involving ferroptosis and lymphocyte infiltration

**Fig. 7:**
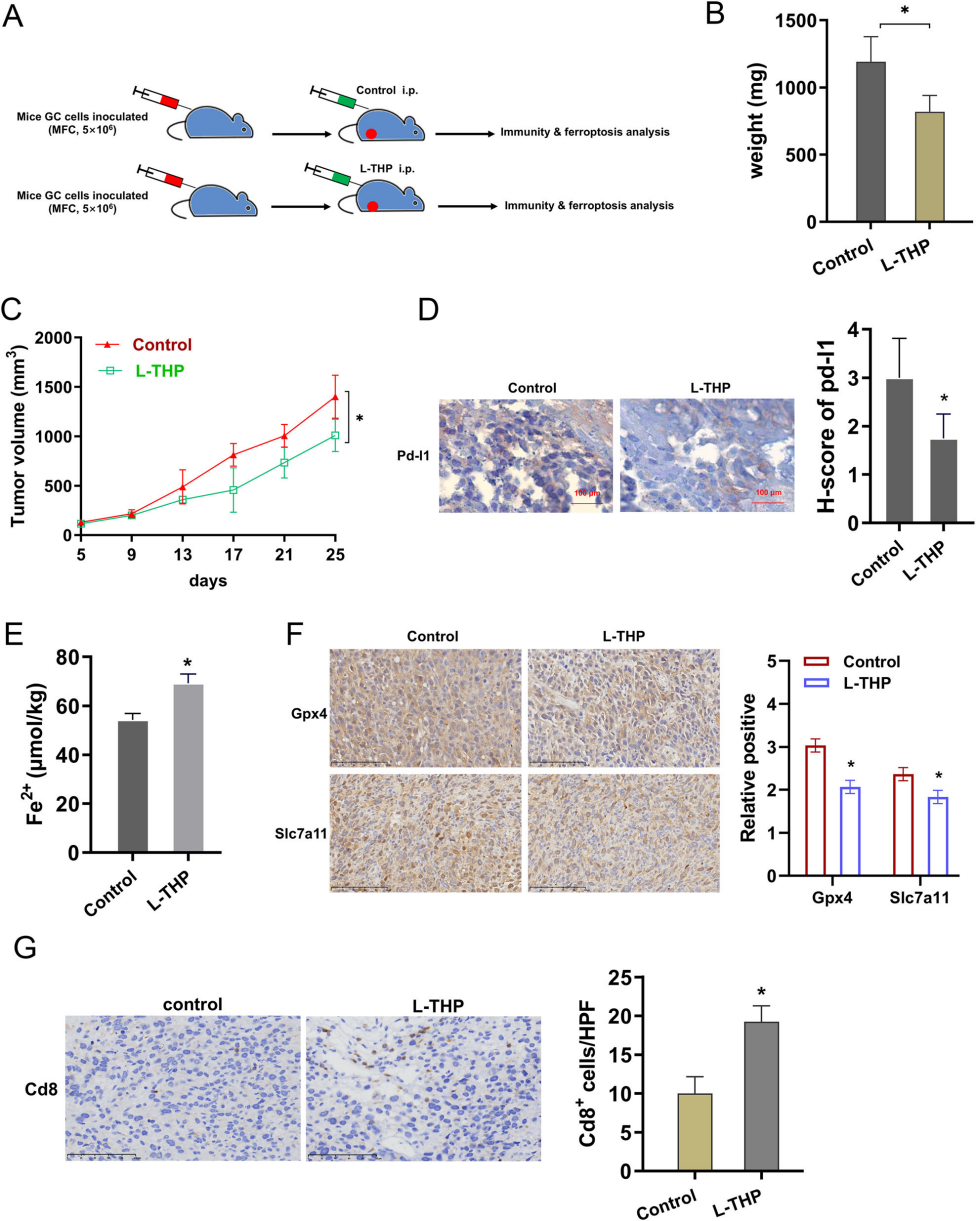
L-THP repressed the GC tumor growth involving ferroptosis and lymphocyte infiltration. **A** In the xenograft assay, the mice GC cells (MFC cells) were inoculated into mice and then the L-THP (150 mg/kg/d, i.p.) was administrated. **B** The weight and **C** volume were calculated to reflect the tumor growth. **D** The immunohistochemical staining of Pd-l1 in L-THP administrated mice and controls. The H-score for Pd-l1 in tissue. **E** Iron (Fe^2+^) level in the tumor tissue. **F** The immunohistochemical staining of Gpx4 and Slc7a11 in tissue samples. The H-score for Gpx4 and Slc7a11. **G** The immunohistochemical staining of Cd8 positive cells tumor samples. The H-score for Cd8 positive cells in tissue. *p < 0.05.

To investigate the role of L-THP in GC tumor growth, an in vivo animal xenograft assay was performed. At the indicated time points, mice GC cells (MFC) were inoculated into mice and L-THP was administrated (Fig. 7A). Results indicated that L-THP repressed the tumor growth, including tumor weight (Fig. 7B) and volume (Fig. 7C). In the resected tissue, the Pd-l1 level was decreased in L-THP group mice as comparing to control (Fig. 7D). For the ferroptosis phenotype, L-THP promoted the iron (Fe^2+^) level (Fig. 7E) and inhibited the Gpx4 and Slc7a11 level (Fig. 7F) in tissue samples. For the tumor immunity, L-THP accelerated the infiltrated CD8^+^ positive lymphocyte in the tumor samples (Fig. 7G). These findings indicate that L-THP repressed the GC tumor growth involving ferroptosis and lymphocyte infiltration.

## Discussion

More evidence is emerging of the roles immune escape on numerous tumorigenesis, and L-THP has shown the tumor suppressor effects. Thus, our work explained the inhibitory effect of L-THP on tumor from CD8^+^ T cells aspect [11]. A series of assays were performed to confirm the function of L-THP and its depth mechanism on GC tumor immunology via FSP1-dependent manner.

**Fig. 8:**
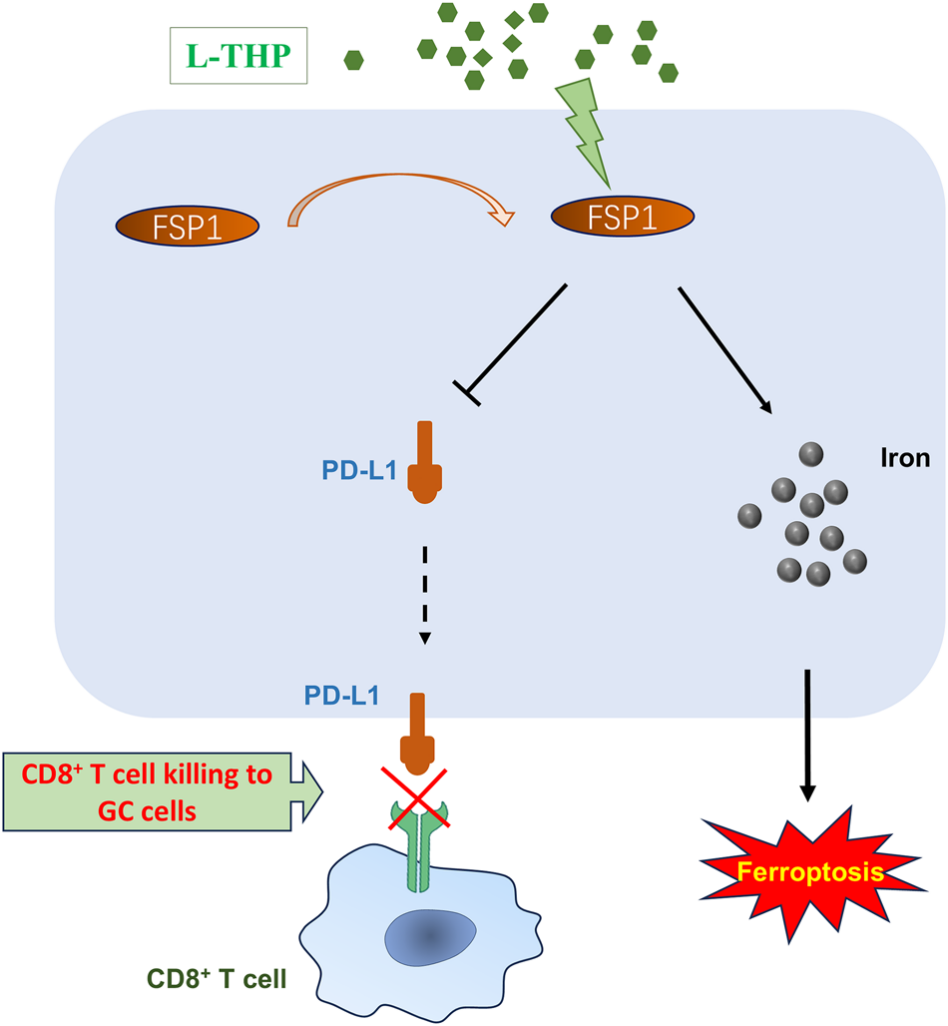
L-THP improves the GC antitumor response and reduces immune escape through FSP1-dependent manner.

Accumulating evidence suggests that L-THP presents anti-cancer activities, through targeting cancer proliferation and invasion in different human cancers. Here, this present study revealed that L-THP accelerated the ferroptosis of GC cells. Besides, L-THP impaired the GC immune escape through enhancing CD8^+^ T cells’ antitumor response. In *vivo*, L-THP repressed the GC tumor growth involving ferroptosis and lymphocyte infiltration. Overall, these data confirmed the antitumor role of L-THP on the GC (Fig. 8).

Given that immunotherapy plays an increasingly important roles in tumor therapeutic schedule, numerous novel discoveries in immunomicroenvironment regulation. Among post-transcriptional regulatory factors, tumor immunology is increasingly being shown to be involved in GC antitumor response [12–15]. For instance, TNM II + III GC with higher intratumoral CXCR5 + CD8^+^ T cell infiltration is more likely to benefit from adjuvant chemotherapy and the infiltrating CXCR5 + CD8 + T cells shows a specific subtype of stem-like CD8^+^ T with effector memory features [16]. Elevated PD-1 expression in CD8^+^ T cells results in immunosuppression and enhances GC lymph node metastasis. Molecularly, IL-8 upregulates PD-1 expression by JAK2/STAT3 signaling activation and inhibits Fbxo38 ubiquitination [17]. Thus, theses literatures indicate that CD8^+^ T-mediated antitumor response plays important function.

Because ferroptosis was driven by the accumulation of in cells, lipid peroxidation was detected for quantitative analysis using flow cytometry confocal imaging. Lipid peroxidation analysis indicated that L-THP treatment increased the lipid peroxidation expression in GC cells. There are emerging evidences that ferroptosis might be implicated in a variety of pathological scenarios, including tumour microenvironment and CD8^+^ T cells effector function. On the other hand, the two influence each other. Weimin Wang et al. (2019) [18] reported that immunotherapy-activated CD8^+^ T cells could enhance the ferroptosis-specific lipid peroxidation in several cancer cells, which increases the ferroptosis and participates in the antitumour efficacy of immunotherapy. Besides, the IFN-γ released from CD8^+^ T cells could downregulate the SLC7A11 expression and glutamate-cystine antiporter system xc- subunits, which impairs the tumor cells’ cystine uptake and promotes cancer cell ferroptosis and lipid peroxidation. Moreover, another finding showed that CD36 mediated tumor-infiltrating CD8^+^ T cells in tumor microenvironment could induce lipid peroxidation and ferroptosis, thereby reducing cytotoxic cytokine production and impairing antitumor ability [19]. Thus, T cell-promoted cancer cells’ ferroptosis is a novel anti-tumour pathway, which is in combination with checkpoint blockade for potential therapeutic.

In conclusion, we demonstrated that L-THP could reduce the GC tumorigenesis involving ferroptosis and antitumor response. Given that CD8^+^ T cells could induce tumor cells’ lipid peroxidation and ferroptosis in tumor microenvironment, this study shows a novel insight that L-THP synergizes cytotoxic CD8^+^ T cell-mediated GC antitumor response and ferroptosis through FSP1 pathway, providing potent evidence for GC immunoregulation.

## Materials and methods

### Cells, culture and L-THP treatment

GC cell lines (AGS, HGC-27 and MKN74) were obtained from American Type Culture Collection (Manassas, VA, USA) and Cell Bank of Chinese Academy of Sciences (Shanghai, China). Cells were cultured in DMEM medium added with 10% FBS (fetal bovine serum, HyClone, Logan, UT, USA) in 5% CO_2_ humidified at 37 °C. For the L-THP treatment, cells were exposed with L-Tetrahydropalmatine (shyuanye, B28183, ≥98%, C_21_H_25_NO_4_, molecular weight: 355.44) at indicated dosage (0 μmol/L, 50 μmol/L, 100 μmol/L).

### Proliferation and migration analysis

To investigate whether L-THP suppress the cell viability, the CCK-8 assay was performed as manufacturer’s protocol. GC cells were seeded in 96-well culture plate. After incubation, CCK-8 reagent (10 μl, Dojindo Japan) was added to wells and the absorbance was detected at 450 nm. For the migration analysis, the migratory ability of GC cells was assessed using a transwell chamber assay following a standard protocol.

### Lipid peroxidation levels

Lipid peroxidation was detected using C11-BODIPY581/591(Invitrogen™ according to the manufacturer’s recommendations. After incubation, lipid peroxidation in the GC cells was fluorometrically detected using fluorescence microscope (Leica Microsystems, Germany) at 470 nm.

### FerroOrange analysis

GC cells were incubated with Hoechst 33342 (1 μg/mL) and FerroOrange (1 μM, cat. F374, Dojindo) and at 37 °C). Fe^2+^ level was analyzed by fluorescence microscopy.

### Transmission electron microscopy (TEM)

GC cells were trypsinized and fixed in glutaraldehyde. Post-fixed was done byosmium tetroxide (1%) in bicarbonate buffer, and then dehydrated in ethanol solutions. Cells were embedded in Poly/Bed 812 resin and then sectioned and captured by transmission electron microscope.

### CD8^+^ T cells preparation

Peripheral blood mononuclear cells (PBMCs) were obtained from healthy donors and then isolated by density centrifugation using Ficoll gradient separation. The cells were washed PBS and suspended in RPMI 1640 media. T cells were stimulated in RPMI containing anti-CD3 (2 μg/m), anti-CD28 (3 μg/ml) and IL-2 (200 U/ml) (Biolegend) for 24 h.

### Cytotoxicity analysis

Stimulated CD8 + T cells acted as the effector cells and GC cells acted as the target cells. The effector/target cells were cocultured at 5:1 ratio. Under standard condition coculture (37 °C, 5% CO_2_) for 7 hours, T-cell mediated cytotoxicity towards GC cells was determined by lactate dehydrogenase (LDH) assay. The culture supernatants were collected and the LDH release in the supernatants was measured using the Cytotoxicity Detection Kit PLUS (Cat no. 04744926001, Sigma-Aldrich) according to the manufacturer’s instructions.

### Enzyme linked immunosorbent assay (ELISA)

The level of IFN-γ and TNF-α were detected using commercial ELISA kits purchased based on the manufacturer provided protocols (RAPIDBIO, CA, USA) in the supernatants of the co-culturing system. The supernatants were collected from co-culturing and incubated with indicated reaction solution. The expression levels of IFN-γ and TNF-α in th co-culturing system supernatants were measured using the commercial ELISA kits as the protocols provided by the manufacturer.

### Surface PD-L1 detection

To detect the surface FSP1 expression, the flow cytometry analyses were performed. The GC cells were centrifuged (1000 g, 5 min) and incubated with PE-conjugated FSP1 antibody (Abcam, ab213524, 1:500 dilution) at 4 °C in dark for 30 min. GC cells were resuspended in FACS washing buffer and then subjected to flow cytometry. Data were analyzed by FlowJ software (TreeStar, Ashland, USA).

### Fe^2+^ detection

For the Fe^2+^ concentration detection, Iron Colorimetric Assay Kit (Applygen, Beijing, China, cat. no. E1042) was performed to quantitatively determine the Fe^2+^ under the ferrozine colorimetric method.

### RT-qPCR

The mRNA levels were detected using quantitative real-time PCR (chain). First, nuclear and cytoplasmic RNA were extracted from GC cells using TRIzol reagent (Invitrogen, Carlsbad, CA, USA) according to the manufacturer’s instructions. cDNA was generated using a PrimeScript RT Reagent Kit (Takara, Dalian, China). qPCR was conducted using a Cham/Q SYBR PCR Master Kit (Vazyme, Nanjing, China) and a 7900 real-time PCR system. The relative expression levels were determined using the 2^−△△CT^ method. Primers: FSP1, Forward, 5’-GTGAGCGGGTGAGCAATCT-3’, Reverse, 5’-CTTGATGCCGGTGCAGAGAA-3’.

### Immunofluorescence

GC cells were fixed by 4% paraformaldehyde and permeabilized by 0.5% Triton X-100, and then blocked. They were then incubated with the anti-PD-L1 (Proteintech, 66248-1-Ig, 1:600), and anti-FSP1 (Proteintech, 20886-1-AP, 1:1000) primary antibodies overnight at 4 °C). The cells were followed by fluorescent secondary antibodies incubation (daylight 488 or 594-labeled, EarthOx) for 1 h. A confocal microscope was utilized for imaging.

### RNA stability assay

GC cells were grown in 12-well plates and collected at the indicated time for RNA extraction. Actinomycin D (Act D, 5 μg/mL, Cell Signaling Technology) was adopted to the cells as treatment. The remaining level of FSP1 mRNA was analyzed by qRT-PCR. The FSP1 mRNA half-life was calculated based on linear regression analysis.

### In vivo assays

The in *vivo* animal experiment was approved by the Animal Care and Use Committee of the Sichuan Provincial People’s Hospital. 4–5-week-old male C57BL mice (weighing between 20 g and 25 g) were purchased from the Laboratory Animal Center and housed for 3–5 days before assays. GC MFC cells (5×10^6^cells/mL, 100 µL) were injected in to flank of the mice to establish subcutaneous tumor xenograft model. To evaluate the effect, L-THP was injected (i.p., 40 mg/kg/day) into mice and administrated every day for 3 weeks. Tumor growth was measured using calipers and determined using 1/2 (length×width^2^).

### Statistical analysis

Data was expressed as Mean ± Standard deviation (SD) from independent three assays. The differences were calculated by one-way ANOVA or Student’s t-test using GraphPad Prism 9.0 software (GraphPad software, La Jolla, CA, USA). The statistical significance was considered when p-value less than 0.05.

### Ethics approval and consent to participate

Written informed consent was obtained from each patient, and the study had been approved by the Ethical Committee of Sichuan Academy of Medical Science & Sichuan Provincial People’s Hospital.
